# Optimized SMRT-UMI protocol produces highly accurate sequence datasets from diverse populations—Application to HIV-1 quasispecies

**DOI:** 10.1093/ve/veae019

**Published:** 2024-03-02

**Authors:** Dylan H Westfall, Wenjie Deng, Alec Pankow, Hugh Murrell, Lennie Chen, Hong Zhao, Carolyn Williamson, Morgane Rolland, Ben Murrell, James I Mullins

**Affiliations:** Department of Microbiology, University of Washington School of Medicine, 960 Republican Street, Seattle, WA 98195-8070, USA; Department of Microbiology, University of Washington School of Medicine, 960 Republican Street, Seattle, WA 98195-8070, USA; Department of Microbiology, University of Washington School of Medicine, 960 Republican Street, Seattle, WA 98195-8070, USA; Department of Pathology, Division of Medical Virology, University of Cape Town and National Health Laboratory Services, Observatory, Cape Town 7925, South Africa; Department of Microbiology, University of Washington School of Medicine, 960 Republican Street, Seattle, WA 98195-8070, USA; Department of Microbiology, University of Washington School of Medicine, 960 Republican Street, Seattle, WA 98195-8070, USA; Department of Pathology, Division of Medical Virology, University of Cape Town and National Health Laboratory Services, Observatory, Cape Town 7925, South Africa; US Military HIV Research Program, Walter Reed Army Institute of Research, 503 Robert Grant Avenue, Silver Spring, MD 20910, USA; The Henry M. Jackson Foundation for the Advancement of Military Medicine, Inc., 6720A Rockledge Drive, Bethesda, MD 20817, USA; Department of Microbiology, Tumor and Cell Biology, Karolinska Institutet, Solnavägen 9, Stockholm 171 65, Sweden; Department of Microbiology, University of Washington School of Medicine, 960 Republican Street, Seattle, WA 98195-8070, USA; Department of Medicine, University of Washington School of Medicine, 960 Republican Street, Seattle, WA 98195-8070, USA; Department of Global Health, University of Washington Schools of Medicine and Public Health, 960 Republican Street, Seattle, WA 98195-8070, USA

**Keywords:** single-molecule-real-time (SMRT) sequencing, unique molecular identifiers (UMI), human immunodeficiency virus, quasispecies, PacBio

## Abstract

Pathogen diversity resulting in quasispecies can enable persistence and adaptation to host defenses and therapies. However, accurate quasispecies characterization can be impeded by errors introduced during sample handling and sequencing, which can require extensive optimizations to overcome. We present complete laboratory and bioinformatics workflows to overcome many of these hurdles. The Pacific Biosciences single molecule real-time platform was used to sequence polymerase-chain reaction (PCR) amplicons derived from cDNA templates tagged with unique molecular identifiers (SMRT-UMI). Optimized laboratory protocols were developed through extensive testing of different sample preparation conditions to minimize between-template recombination during PCR. The use of UMI allowed accurate template quantitation as well as removal of point mutations introduced during PCR and sequencing to produce a highly accurate consensus sequence from each template. Production of highly accurate sequences from the large datasets produced from SMRT-UMI sequencing is facilitated by a novel bioinformatic pipeline, Probabilistic Offspring Resolver for Primer IDs (PORPIDpipeline). PORPIDpipeline automatically filters and parses circular consensus reads by sample, identifies and discards reads with UMIs likely created from PCR and sequencing errors, generates consensus sequences, checks for contamination within the dataset, and removes any sequence with evidence of PCR recombination, heteroduplex formation, or early cycle PCR errors. The optimized SMRT-UMI sequencing and PORPIDpipeline methods presented here represent a highly adaptable and established starting point for accurate sequencing of diverse pathogens. These methods are illustrated through characterization of human immunodeficiency virus quasispecies in a virus transmitter-recipient pair of individuals.

## Introduction

Viral quasispecies are populations of genetically related non-identical viruses—typically ribonucleic acid (RNA) viruses—with the potential to facilitate adaptation to a changing host environment and thus contribute features beneficial to the survival of the overall population within a host (Domingo and Schuster 2016; Domingo and Perales 2019; Sanjùan and Domingo-Calap 2021). Viral quasispecies have traditionally been examined by Sanger sequencing of individual viral templates; yet such an approach is low throughput (usually limited to a few dozen viruses) and cost-intensive, as limiting dilution of templates is required for highly accurate sequence derivation (Simmonds et al. 1990). While next-generation sequencing provides a high-throughput alternative, the short-read lengths, e.g. using the Illumina platform, typically prevent studies of linkage across genes much longer than sequence reads (150–60 bp) (Goodwin, McPherson, and McCombie 2016; Ravi, Walton, and Khosroheidari 2018). Pacific Biosciences (PacBio) single-molecule real-time (SMRT) (Ardui et al. 2018) and Nanopore (Wang et al. 2021) sequencing technologies are both third generation long-read, high-throughput sequencing platforms capable of producing read lengths in excess of 10 Kb (Pollard et al. 2018). However, errors inherent in these technologies and introduced during polymerase-chain reaction (PCR) can persist within the population of sequenced molecules and prevent accurate assessment of viral quasispecies. Furthermore, PCR-mediated recombination (chimera formation) (Judo, Wedel, and Wilson 1998) can erroneously link viral mutations and can be mistaken for viral recombination *in vivo*, thus obscuring accurate quasispecies assessment. The PacBio SMRT platform was chosen for sequencing here because of the lower error rate derived from circular consensus sequences (CCS) (Wenger et al. 2019) compared to current Nanopore approaches (Weirather et al. 2017; Delahaye, Nicolas, and Andrés-León 2021).

Incorporation of molecular tags composed of random sequences (typically 6–12 nt), referred to as primer IDs (Jabara et al. 2011) or unique molecular identifiers (UMIs) (Kivioja et al. 2011), into complementary DNA (cDNA) primers used in the first step of RNA virus genome amplification tags every cDNA molecule with a random sequence that is preserved during subsequent PCR amplification. Importantly, the number of cDNA templates that are then amplified can be accurately determined by the number of unique UMIs recovered (Ardui et al. 2018). By collecting CCS reads (hereafter referred to as ‘reads’) with the same UMI (hereafter referred to as UMI families), then generating a consensus, PCR and sequencing errors can be removed to accurately estimate the sequence of each original cDNA template. A single UMI (sUMI) alone cannot always identify products that incurred template switching during PCR (often referred to as recombination). However, incorporating a second UMI into the opposite strand labels each viral template with a unique combination of UMI sequences, one on either end of the molecule (duplex UMI or dUMI) (Schmitt et al. 2012). By comparing the frequencies of different UMI combinations and comparing UMI sequences between families, reads representing molecules that ‘recombined’ during PCR can be identified. However, drawbacks to the dUMI approach exist, such as loss of sample material during multiple purification steps necessary to remove excess primers and fixation of errors during second strand synthesis. Thus, we sought sUMI sample preparation conditions resulting in low levels of recombination with accuracy approximately equivalent to dUMI.

As part of our workflow, a bioinformatics pipeline was developed to take CCS as input and produce tables of all observed UMI combinations, as well as consensus sequences prepared from the sUMI or dUMI read subsets of each family. This allowed for direct comparisons between consensus sequences from sUMI and dUMI methods.

After optimizing laboratory methods for sUMI, a computational pipeline named Probabilistic Offspring Resolver for Primer IDs (PORPIDpipeline) was created. This pipeline filters by read length and quality, separates sequences by sample ID and then by UMI, and removes: UMI families likely to be ‘offspring’ generated by errors from real UMI families; UMI families characterized as heteroduplexes; UMIs not matching the expected length; UMI families derived from fewer than five reads. Consensus sequences are generated for each remaining UMI family, and sequences with consensus agreement less than 0.7 at any base position are discarded. Downstream processing includes alignment and trimming against reference panels, identifying likely contamination, both from common lab strains and cross-contamination between samples prepared together, and providing hypertext markup language (HTML) reports with various quantity and quality metrics, as well as visualizations of phylogenies, alignments, apolipoprotein B mRNA editing enzyme, catalytic polypeptide (APOBEC)-mediated hypermutation, and more.

The results shown here, taken either as guidelines or as an optimized sUMI workflow, combined with the PORPIDpipeline represent a streamlined and generalized sequencing approach, which can be highly adaptable and is anticipated to help advance the study of pathogens that develop intra-host genetic diversity during infection as well as studies of diverse organism communities in general. An example of the use of SMRT-sUMI is provided through analysis of quasispecies present in a human immunodeficiency virus (HIV) transmitter-recipient pair.

## Results

### Preliminary assessment of sequence quality

Preliminary experiments assessed the accuracy of PacBio CCS compared to the Sanger method. First, a mixture of fifty-four 1.2 kb amplicons previously sequenced by Sanger single genome amplificaiton (SGA), mixed at different concentrations and differing by as little as 1 bp were sequenced without UMI. We recovered each of the exact sequences after SMRT sequencing, no sequences were found with additional mutations, and sequences were recovered at roughly the correct ratios, confirming the accuracy of the PacBio platform in our hands. A series of additional studies were performed using plasma from HIV-infected persons as starting material and previously sequenced by Sanger SGA to directly compare the two methods. In all, both Sanger SGA and the SMRT-sUMI sequencing methods described later were conducted on more than 100 samples over a 2.5 kb region including the HIV gag gene and the same 3.1 kb fragment including the HIV env gene used to illustrate the technique in this study. Examples of such data are shown in Supplementary Fig. S1.  Supplementary Fig. S1A shows an early study of three individuals in which plasma was used as starting material for both methods, and few SMRT-sUMI sequences were loaded on the instrument. Excellent concordance between SGA and SMRT-sUMI sequences was observed, as indicated by sequence intermingling in all branches of the trees, and the similarity in overall pairwise sequence diversity is shown in the table inset. To further assess the reproducibility and consistency of the SMRT-sUMI method, this time using the same cDNA preparation, we repeated the PCR and sequencing of viral genome amplicons using SMRT-sUMI. Supplementary Fig. S1B shows a phylogenetic tree with the original SMRT-sUMI sequence names in black and those resulting from repeated PCR and SMRT-sUMI sequencing in pink. The samples were intermingled in the tree, and the overall diversity was the same for each replicate. In summary, these results show the SMRT-sUMI method to accurately reproduce sequence data when compared to the gold-standard of amplicon sequencing using SGA and Sanger methods and also to give consistent and reproducible results across experiments.

### Optimization of laboratory protocols

**Figure 1.**
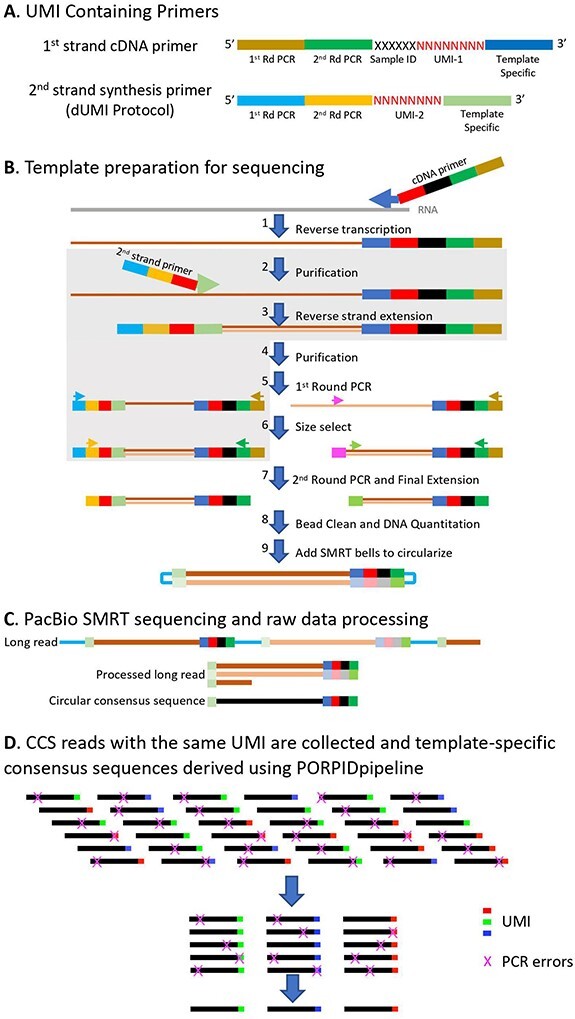
Overview of the viral genome sequencing process. A. cDNA synthesis is conducted using a library of primers with the following structure (reading from the 5ʹ end of the primer): Universal reverse first and second PCR primers, a 6 nt sample ID, an 8 nt random sequence corresponding to the universal molecular identifier (UMI) and a viral template-specific sequence at the 3ʹ end. The second strand synthesis primer is identical except that it lacks the 6 nt sample ID. B. Overview of the sequencing template generation process. Following cDNA synthesis (1), unused primers are removed by bead cleaning (2/4). For sUMI samples, this step (4) is followed by first round PCR (5). For dUMI samples (unique processes indicated by the grey box), a second strand primer containing another UMI is introduced by a single round of PCR (3), followed by another round of bead cleaning to remove unused primers (4). The first round PCR is conducted using the first round universal reverse primer described in Panel A, and either a forward HIV template-specific primer (magenta) for sUMI or a universal forward primer for dUMI (light blue) (5). PCR products below the expected size are then removed using bead purification or the Blue Pippin instrument (6). A second round of PCR is then conducted followed by a single-cycle final extension with new reagents and inner universal reverse and HIV-specific forward primers for sUMI (green) or inner universal forward (yellow) for dUMI (7). PCR primers are removed by bead cleaning and the products quantified (Qubit) and reactions pooled (8). SMRTbell barcoded adapters are ligated onto the ends of the PCR fragments to create a circular molecule (9). C. Illustration of PacBio sequence generation. A sequencing primer is annealed to the SMRTbell template and DNA polymerase is bound to the complex. This polymerase-amplicon-adaptor complex is then loaded into zero-mode waveguides (ZMWs) where replication occurs, producing nucleotide-specific fluorescence upon incorporation into DNA. The polymerase repeatedly replicates the circularized strand, producing one long read with randomly distributed errors. Post-run, the sequences are processed on the instrument—SMRTbell sequences are trimmed away, single-molecule fragments are aligned, and a CCS is generated. D. The final step in sequence generation for sUMI samples is the identification of CCS with the same UMI and generation of a template-specific consensus sequence using the PORPIDpipeline.

Figure 1 presents the overall workflow of the sUMI and dUMI methods, both of which consist of primer design, template preparation, SMRT sequencing and data processing, identification of reads with the same UMI, and derivation of consensus sequences for each viral template. As shown in Fig. 1A, UMIs can either be applied to cDNA alone (sUMI) or to both first strand cDNA and second strand synthesis products (dUMI). Figure 1B includes the initial reverse transcription step of cDNA synthesis from RNA templates; errors introduced at this step cannot be corrected at any point downstream. cDNA synthesis errors accumulate according to the fidelity of the chosen reverse transcriptase and the relevant reaction conditions (e.g. reported error rates for Moloney murine leukemia virus-based reverse transcriptases range from one per 15.5 kb (Arezi and Hogrefe 2007) to one per 27 kb synthesized (Potter, Zheng, and Lee 2003)). cDNA products are purified following the cDNA synthesis step to remove unincorporated primers (Fig. 1B). In the case of dUMI, the purified cDNA is subjected to second strand synthesis with a primer containing a second UMI (steps shaded in gray in Fig. 1B). The resulting double-stranded DNA (dsDNA) product is again purified to remove unincorporated primers. When only sUMI are to be employed, or after second strand synthesis in the dUMI protocol, the purified products are subjected to nested PCR, in order to generate sufficient material for addition of SMRT bells (Travers et al. 2010) for sequencing. As heteroduplexes formed in the last cycle of PCR can result in reads containing artifactual UMI sequences, their formation is avoided by performing a single cycle of synthesis and extension (final extension) using fresh reagents to bias utilization of PCR primers as opposed to incomplete PCR products serving as primers (Laird Smith et al. 2016). Without this step, many reads will fail the heteroduplex filter in the first steps of the PORPIDpipeline (discussed later) and be discarded, leading to lower read depths for each UMI family.

Samples were pooled into sets with non-overlapping IDs and sequenced on the Sequel II instrument from PacBio. After PacBio CCS (Travers et al. 2010) (Fig. 1C), only reads with a Quality (Q) score ≥20 and a size roughly equivalent to our sequenced amplicon were retained. While prior PacBio instruments exhibited loading bias, increasing the frequency of shorter amplicons, with current Sequel II instruments, reagents and software, roughly equivalent CCS recovery of 2.5 kb to 6 kb amplicons were obtained when loaded onto the same plate at equimolar ratios. In our optimized workflow, a consensus of sUMI labeled templates is generated (Fig. 1D) to remove additional PCR errors.

### dUMI sequencing

The dUMI sequencing approach results in a consensus sequence derived from each family of reads with the same combination of UMI sequences. Use of dUMI and the requirement for consistent UMI on each strand to perform consensus generation lowers the error rate to approximately 10^–9^/base incorporated during PCR amplification (Schmitt et al. 2012). A second advantage of using dUMI is that recombinant molecules are readily identified by detection of discordant dUMI and excluded; such recombinant molecules are produced by template switching when incomplete products act as primers during the PCR.

Table 1 illustrates a scenario in which each member of a family of reads shares the same UMI-1 sequence (applied during first strand cDNA synthesis); yet the UMI-2 sequences (applied to the opposite end of the molecule during second strand synthesis of the dUMI protocol) differ across the reads. In this case, the most prevalent dUMI combination (Rank 1) is inferred to represent the original template, and less frequent combinations are inferred to represent combinations introduced by recombination, or PCR or sequencing errors accumulating within the UMI-2 sequence.

**Table 1.** Family of reads sharing same UMI-1 sequence (sUMI family).

UMI-1	UMI-2	Family size	Rank	Levenshtein distance*	Matches UMI-2 from different family
CCGTGGAT	CAATCACC	180	1		
CCGTGGAT	CAATCCACC	1	2	1	No
CCGTGGAT	AATTCACC	1	3	2	No
CCGTGGAT	AATGATGT	1	4	5	No
CCGTGGAT	GGGAACCC	1	5	5	Yes
CCGTGGAT	GTGGTACT	1	6	6	No
CCGTGGAT	TAAGATGG	1	7	6	Yes

* Distance (Levenshtein 1966) from Rank 1 UMI-2 to other UMI-2 sequences observed.

### Optimization of the sUMI protocol

A disadvantage of the dUMI approach, specific to the use of single-stranded templates such as cDNA, is that errors generated during second strand synthesis would be fixed, as only the molecules with the second strand synthesis primer would be amplified in subsequent cycles. Based on published error rates of the PCR enzyme used in our studies and a 3 kb amplicon, this would correspond to one error in every 5–6 starting templates (TakaraBio). A second disadvantage is that significant material loss can occur during bead purification to remove primers, a process required after both first and second strand synthesis reactions. Sample preparation conditions were therefore sought to minimize the rate of recombination and samples loss to allow use of the sUMI workflow without specific identification of recombinant reads via detection of discordant dUMI. To assess different reaction conditions, an initial experiment of 33 samples were prepared for sequencing using the dUMI approach with the goal of sequencing 100 total cDNA templates. The number of estimated input templates (e.g. 25, 50, 100) per PCR, purification method of the first round PCR products, and PCR cycle number were varied to test different combinations. First round PCR reactions from each sample were divided and subjected to size selection with either AMPure XP beads (Beckman Coulter) or the Blue Pippin gel electrophoresis instrument (Sage Science) and compared to fractions that were subjected to identical dilutions but not purified. These separate preparations were amplified in a twenty-two-cycle second round PCR. To test whether lower PCR cycle numbers at high-template input could help to reduce recombination rates, the Blue Pippin purified fractions from the 100 template reactions were separately amplified in second round PCRs consisting of 20 cycles. All second round preparations were then carried forward in an identical manner for subsequent purification, PCR with Index primers, SMRT sequencing, and analysis. Subsequently, reaction conditions were assessed in a further sixteen samples across a range of different amplicons.

For the studies described here, five amplicons ranging in size from 2.1 kb to 5.2 kb were derived from virion-associated RNA from simian immunodeficiency virus (SIV) or HIV or from Kaposi Sarcome Herpes Virus (KSHV) DNA from the 56 different sample preparations (Supplementary Table S1). These studies yielded 8,145 sUMI families that passed all UMI filtering steps of the pipeline and contained at least five members to generate a consensus, and hence were deemed ‘likely real’. Next, examining the UMI-2 sequences found in these sUMI families resulted in identification of 73,402 unique dUMI families. The origin of the large increase in dUMI compared to sUMI families is illustrated in Table 1, where reads with the same UMI-1 (sUMI family) are found to contain seven different UMI-2 sequences (with each combination representing a distinct dUMI family). Levenshtein distances (LDs) (Levenshtein 1966) were calculated for every distinct UMI-2 (contained within the second strand synthesis primer) sharing the same UMI-1 (contained within the cDNA primer). These distances count the fewest number of substitutions, insertions, or deletions necessary to create each observed UMI-2, relative to the most prevalent (Rank 1) UMI-2 in that family. dUMI combinations with distances greater than 1 or 2 are more likely to result from recombination events, as the risk of accumulating greater than two errors via PCR or sequencing error within the relatively short 8 bp UMI region is very low and UMI-2 heteroduplexes should be rare after filtering.

**Figure 2.**
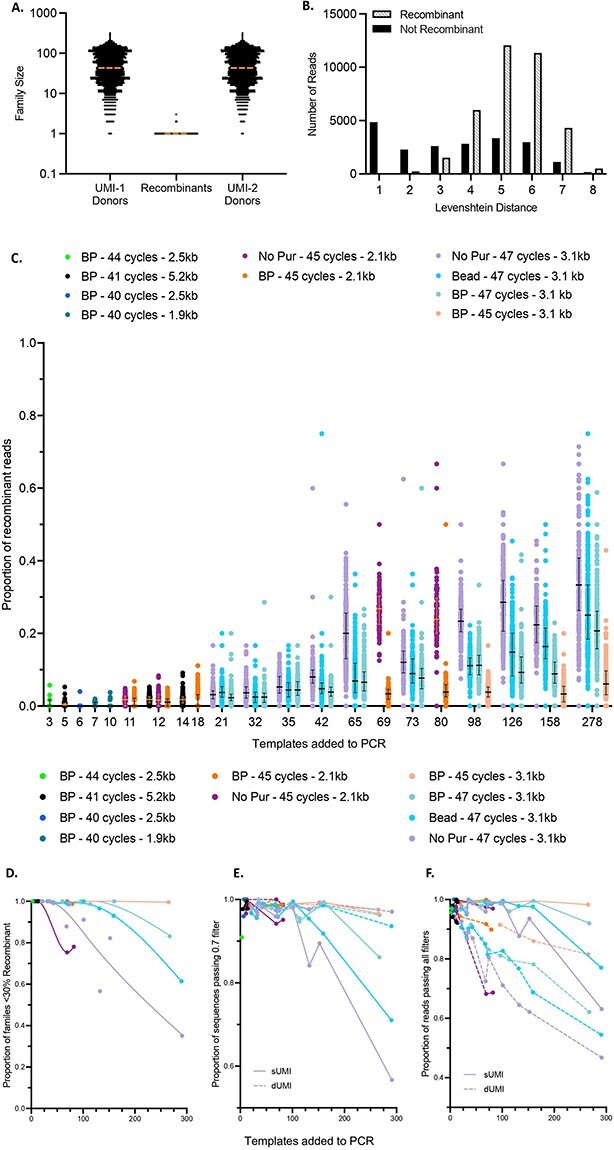
Identification of recombinant reads and conditions that limit their effect. A. Family size is indicated by a dot for each dUMI family. Families judged to be the result of recombination are indicated in the center, while the families that donated a UMI are indicated on either side. Dashed horizontal orange lines indicate the medians. B. LDs are assigned to each read in a dUMI family. The total counts of all reads judged to be recombinant or not are indicated for each LD. C. Proportion of recombinant reads for each sUMI family, grouped by average template input into PCR, where each dot represents an individual sUMI family, colored by purification method, total PCR cycle number, and amplicon size. D. The proportion of sUMI families in each sample containing fewer than 30 per cent recombinant reads at different template inputs into PCR. E. Proportion of sequences that pass the 0.7 minimum agreement filter in the PORPIDpipeline at different template inputs into PCR. F. Proportion of reads passing all filters in the PORPIDpipeline at different template inputs into PCR.

To identify which UMI combinations within each sUMI family were the result of recombination during PCR, the rank >1 UMI-2 sequences from each family were compared to the Rank 1 UMI-2 sequences from all other families in the same sample. Conservatively, any rank >1 families with a UMI-2 that matched a Rank 1 family were judged to result from recombination. As we required an exact match between UMI-2 sequences to classify a family as recombinant, the numbers of families noted as recombinants are likely an underestimation, as any PCR error in the UMI-2 after a recombination event would appear as a unique UMI. Nearly all Rank 1 families were found to donate both UMI-1 and UMI-2 sequences to recombinant reads. In most cases, the size of the recombinant family was 1 (and hence omitted from consensus sequence generation), while the size of the Rank 1 families where the recombinant was derived from was much larger (Fig. 2A); the median size of Rank 1 families that donated a UMI sequence to a recombinant was 43, while the median of those that did not was 9.

As anticipated, dUMI reads with UMI-2 sequences most similar to the Rank 1 family (LD 1 or 2) were not frequently judged to be recombinants, owing likely to their origin as point mutations in the PCR amplification process. In contrast, ∼75 per cent of all dUMI reads with LD >3 appear to be recombinants (Fig. 2B; here, the number of reads reflecting the combined data from all UMI families are plotted). We judged that it was unlikely that many of the latter resulted from an accumulation of 4+ changes to the original UMI-2 sequence, but more likely resulted from a recombination event. The low prevalence of LDs 7 and 8 in recombinant and non-recombinant read collections likely results from the low chance of any two compared UMIs having a different base in all but 1 (LD = 7) or in every position (LD = 8).

Next, PCR input template number and purification of first round PCR products were optimized to limit recombinant yield. Initial experiments focused on a 3.1 kb region of HIV, which encompasses the entire HIV env gene, starting in the rev gene and ending in the nef gene. This allows the study of linkage between mutations across the entire targeted gene of interest (the env gene). While we aimed to add 25, 50, or 100 templates to each PCR based on estimates from limiting dilution PCR, the number of templates recovered varied from 21 to 278. To give sufficient sequence depth for comparisons of consensus sequences between sUMI and dUMI methods, a combined total of at least 100 estimated templates were targeted for each. As a result of varying template input, the number of first round PCR reactions conducted per sample varied from 1 to 10. To validate the findings from the 3.1 kb env experiment, sixteen additional samples were examined from four additional amplicons: nine from a 2.1 kb region of SIV, plus Blue Pippin purified samples from three 5.2 kb 3ʹ halves of the HIV genome, and four samples spanning either the KSHV repeat regions IR1 (2.5 kb) or LANA (1.9 kb). The template inputs for these additional samples ranged from 3 to 82, lower than the 3.1 kb samples primarily due to reduced PCR efficiencies for the larger fragments and sample limitations in the others (Supplementary Table S1).

Smaller, off-target PCR products are frequently generated when amplifying large amplicons. Prevalence of these off-target products varied by sample and PCR conditions but without effective removal can prevent efficient amplification and recovery of larger, targeted amplicons. Additionally, off-target products present in the final sequencing mixture can reduce sequencing depth of the target amplicon. By purifying the first round PCR products, some off-target products are removed, and subsequent PCR rounds efficiently amplify the remaining PCR products. When evaluating and selecting a purification method, a somewhat wide range of molecular sizes should nonetheless be captured to avoid loss of molecules of interest that may have suffered large internal deletions or insertions *in vivo*. For example, Supplementary Fig. S2 shows an off-target band of 2 kb. Sequence analysis showed that the 2 kb bands corresponded to truncated molecules created by a cDNA mispriming event (due to a primer 3ʹ end homology within the target amplicon) resulting in the loss of about 1000 bases near the 3ʹ end of the amplicon. If truncated amplicons or those with internal deletions were of interest, a wider capture window, purification with AMpure beads, or no purification at all should be used to ensure all molecules of interest are sequenced. Here, two purification methods were compared to unpurified PCR products—size selection using the Blue Pippin instrument and AMPure XP beads.

Blue Pippin purification resulted in substantially more efficient removal of smaller PCR products and somewhat greater yield of the desired product than purification with beads, each compared to unpurified fractions (Supplementary Fig. S2). Figure 2C shows the proportion of recombinant reads found in each sUMI family as a function of the number of templates in each first round PCR and the PCR product purification method. At lower input numbers (≤50), relatively low fractions of recombinant reads were observed without purification. Increasing initial template number per first round PCR resulted in benefits from purification. Furthermore, reduction of PCR cycles reduced recombination in the Blue Pippin fraction to greater effect, resulting in rates comparable to much lower template inputs. This indicates that using the fewest PCR cycles that still result in sufficient DNA yield for sequencing is ideal. It should be noted, however, that in most cases multiple parallel second round PCRs are required to generate the DNA mass required for sequencing. While unpurified samples generally have a greater proportion of recombinant reads, target bands on 1 per cent agarose gels are stronger in purified samples using the same cycle number (Supplementary Fig. S2), suggesting that recombination rates are influenced substantially by the prevalence of smaller PCR products in the mixture. To perform PCR with low recombination rates, a balance must be reached between first round PCR purification method, template input, and cycle number. If a restricted size window is acceptable, Blue Pippin purification could be used to remove all off-target bands and ∼275 templates added to each PCR reaction when using forty-five total cycles (Fig. 2C). However, if it is preferable for all PCR molecules to be amplified and sequenced without first^t^ round PCR purification and forty-seven total cycles are used, template input should be limited to less than 50 per PCR to achieve similarly low levels of recombination. In either case, greater numbers of sequences can be obtained by increasing the number of first round PCRs. If e.g. 500 sequences were desired, 2 PCRs with Blue Pippin purification and 45 cycles could be performed (2 × 250 template input), while 10 PCRs would be required if performing no purification and using 47 cycles (10 × 50 template input). In both cases, the desired total templates and purification methods can be used with similarly low levels of PCR recombination.

To facilitate the use of the sUMI workflow, strict methods were developed within a computational pipeline (PORPIDpipeline, discussed later) to remove families with sequence variation great enough to impact the consensus. A sUMI family is discarded by the pipeline if the minimum agreement at any position in its alignment was <70 per cent (hereafter referred to as the 0.7 filter). This removes families with any positions of low confidence in base calls and results in only sequences passing the filter, which have high agreement at every position; critical in studies of low frequency mutations. Positions of low agreement/confidence can result from variation introduced by PCR recombination or by errors introduced when the original template is first copied, where the error would be present in only one of the first two DNA strands (rate ∼ 0.5). If an error occurs during the first cycle of PCR (when both strands are copied), three of the four strands (rate ∼ 0.75) would have the correct base. Errors in successive PCR cycles would result in greater agreement levels. Ignoring the frequency fluctuations due to stochastic template resampling, the 0.7 filter thus removes any families with second strand synthesis errors and/or which underwent sufficient recombination to drop agreement levels below 0.7. In our dUMI data, we observed second strand errors (agreement near 0.5) taking place in about 1.7 per cent of our families. Interestingly, this is about 10× lower than what would be expected when calculating from the error rate of the PCR enzyme (6.2 × 10^−5^) provided by the manufacturer (Takara Bio), though similar to the error rate found in another study using PacBio sequencing (8.4 × 10^−6^) (Potapov, Ong, and Kalendar 2017). dUMI data were used to estimate the number of sUMI families that would pass the cutoff by plotting the number of families containing <30 per cent reads identified as recombinant for different preparation conditions (Fig. 2D). Here, we assumed that each recombinant read would drop agreement in the sUMI family, so this should represent the minimum proportion of families expected to pass the 0.7 filter. In reality, a read may recombine with another identical read, so while the dUMI analysis may identify this read as a recombinant, it would not impact the agreement at any position. Thus, in samples with little genetic diversity, recombination could take place with minimal impact on agreement or consensus sequence. Figure 2D shows that when no purification was performed, the fraction of sUMI families with <30 per cent recombinant reads decreased rapidly with increasing template input per PCR. In contrast, when using either of the two purification methods, the fraction of sUMI families with <30 per cent recombinant reads usually remained stable at ∼95 per cent until approximately 100 templates were added to the PCR. At template inputs >100, fewer of the sUMI families from the bead-purified samples had <30 per cent recombinant reads compared to those purified by Blue Pippin, especially those with reduced cycle numbers, consistent with the results from the CCS read quantitation shown in Fig. 2C.

To help understand how the recombination observed in the UMI analysis impacted the accuracy and filtering of sequences calculated from sUMI compared to dUMI read collections, consensus sequences were generated using both methods and subjected to the 0.7 filter. sUMI consensus sequences are derived from all reads in a UMI-1 family, while only the reads from the most frequent UMI combination (Rank 1) are used to create the corresponding dUMI consensus sequence. Aside from errors introduced during second strand synthesis, dUMI consensus sequences are therefore believed to be the most accurate. While Fig. 2D estimated the minimum number of families expected to pass the 0.7 filter based on identification of recombinant dUMI reads, Fig. 2E shows the number of consensus sequences from these families that did pass the 0.7 filter. This is a direct comparison of how reads identified as recombinant in the dUMI data influenced the agreement in their sUMI families. As predicted from the data in Fig. 2D, many of the sUMI sequences from template inputs greater than 100 were discarded by the 0.7 filter, with unpurified samples suffering the greatest loss. However, the number of sUMI sequences passing the 0.7 filter was greater at template inputs above 100 than predicted from Fig. 2D, with the greatest difference seen in unpurified samples. Because unpurified samples have the greatest number of recombinant reads, but more sequences than expected pass the 0.7 filter, it is clear that many of the recombinant reads have no effect on agreement within their sUMI family. As expected, nearly all dUMI sequences pass the 0.7 filter (mean rate = 0.98, range 0.91–1.00). Twenty-six of the 153 dUMI sequences that failed the 0.7 filter (of a total of 7840 sequences) were investigated after sequence alignment, and at each site of discordance was a position with two bases present in similar frequencies, indicative of an early cycle PCR error (81 per cent), or a result of homopolymer length variation (19 per cent). These types of errors cannot be corrected by either dUMI or sUMI methods.

Figure 2F indicates the proportion of reads remaining after filtering. Because sUMI families contain reads with rank >1, while dUMI families do not, the greater number of reads retained in the sUMI fraction represent rank >1 reads that do not cause the sUMI family to fail the 0.7 filter. This again shows that many recombinant reads have no effect on agreement within their sUMI family and highlights the ability of the 0.7 filter to only remove sequences with recombination or error levels great enough to create uncertainty in the sequence.

Matched (same UMI1) sUMI-dUMI consensus sequences from all preparation types were compared, and an error rate of 1 in ∼394,500 bp was observed. Samples with fewer than 100 templates that were also purified offered the lowest per-sequence and per-base error rates (Table 2). Ten (of 5,555) families had discordant sUMI and dUMI sequences in samples with <100 input templates. In all cases, discordance appeared to be caused by UMI collisions (i.e. two different cDNA molecules receiving the same UMI sequence by chance) or an error during second strand synthesis resulting in sites with similar nucleotide frequencies, while none had errors due to PCR recombination. Four total UMI collisions were observed in our dataset, whereas ∼10 were expected based on the logic of the birthday paradox when calculating the probability of two RNA templates receiving identical UMIs from the total of 65,536 unique UMI combinations (4^8^) in our samples with an average of ∼160 starting RNA templates. In contrast, in sequences from reactions with >100 templates, 25/34 discordant families appeared to result from PCR recombination instead of UMI collisions or errors during second strand synthesis. In these families, reads with the most prevalent UMI-2 sequence were outnumbered by reads from all other UMI-2 sequences, which changed the majority nucleotide at that position. This demonstrates how artifactual consensus calls can occur when conditions allow high rates of PCR recombination.

**Table 2.** Discordant (error) rates using UMI and 0.7 minimum agreement filter.

A. All Families					
			Discordant:		
Sample type	Families	Bases	Sequences	Rate/sequence	Rate/base
No purification	2402	6,615,163	26	0.0108	5.90E−06
Purified 1st rd PCR	5438	16,661,943	18	0.0033	1.20E−06
≥100 templates	2285	6,699,534	34	0.0149	5.82E−06
<100 templates	5555	16,577,572	10	0.0018	1.21E−06
≥100 templates or no purification	4056	11,508,415	40	0.0099	4.78E−06
<100 templates and purified	3784	11,768,691	4	0.0011	3.40E−07
Families passing 0.7 agreement filter					
			Discordant:		
Sample type	Families	Bases	Sequences	Rate/sequence	Rate/base
No purification	2229	6,109,155	1	0.0004	1.64E−07
Purified first rd PCR	5264	16,134,650	1	0.0002	6.20E−08
≥100 templates	2036	5,955,632	2	0.0010	3.36E−07
<100 templates	5457	16,288,173	0	<0.00018	<6.14E−08
≥100 templates or no purification	3768	10,658,191	2	0.0005	1.88E−07
<100 templates and purified	3725	11,585,614	0	<0.00027	<8.63E−8

Knowing that many families contained high levels of PCR recombination, we next restricted the sUMI-dUMI sequence comparisons to all matched sequences passing the 0.7 filter. In doing so, the per base error rate was reduced to 1 in ∼1.1 × 10^7^ bp. No differences were found between matched sUMI and dUMI sequences that passed the 0.7 filter in samples with fewer than 100 templates in the PCR (Table 2). This emphasizes the critical role the 0.7 filter plays in reducing errors when utilizing the sUMI method.

### Automation of data processing

**Figure 3.**
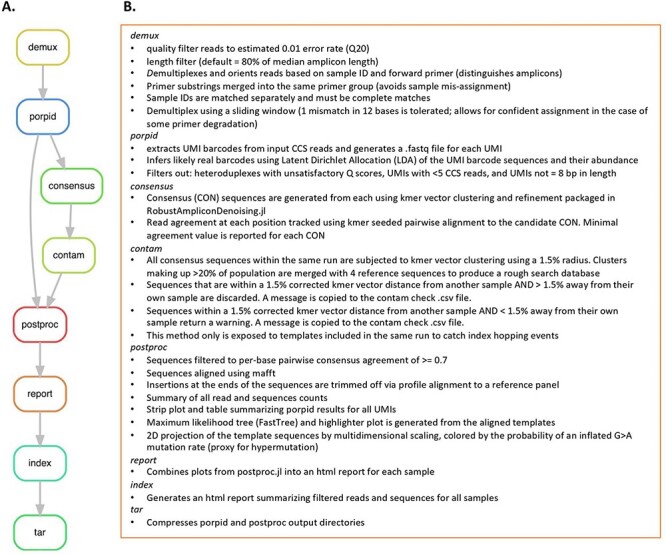
Overview of PORPIDpipeline. A. Flowchart illustrating flow between each rule within PORPIDpipeline and a brief description of each in B. See Methods for detailed descriptions. Only rules demux, porpid, and consensus were present in the pipeline used for analysis of the dUMI preparations.

**Figure 4.**
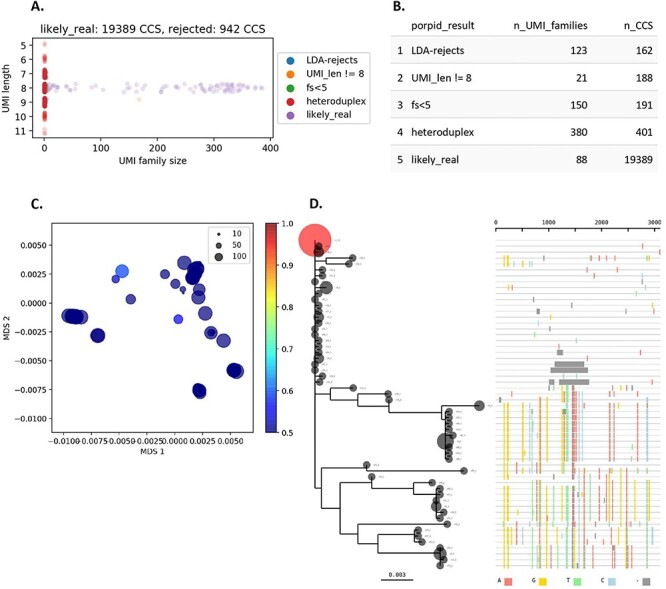
Elements of a PORPIDpipeline report. Panels A and B show elements of the results of PORPID processing, Panels C and D show results of post-processing steps of single-template consensus sequences. All plots shown here are examples and do not contain data from the samples used in this study. A. UMI stripplot. UMI family size (the number of individual reads with a given UMI) is shown as a function of the UMI length determined by the PORPIDpipeline. Only ‘likely_real’ UMIs are kept for downstream analysis. Those removed include the ‘LDA-rejects’ that are likely generated by errors in the UMI sequence, ‘fs<5’, which indicates sequencing depth was under 5 CCS reads and considered too low for consensus analysis, and UMIs flagged as ‘heteroduplex’ when the set of reads had a signature drop in quality in the UMI region indicative of a superimposed signal of two different UMI sequences during circular consensus generation. Some UMIs of length other than 8 pass all other criteria, but are still excluded from analysis (‘UMI_len != 8’). B. UMI family and CCS read totals are provided for each type of PORPID result post-processing of single-template consensus sequences. C. Multidimensional scaling (MDS) of template sequences and G > A APOBEC model. Classical MDS was used to represent all pairwise distances between template sequences in 2D space. Individual points are scaled by their family size (see inset key). A Bayesian model for APOBEC hypermutation is run on the single nucleotide substitution matrix between the global consensus and query sequence and estimates the overall mutation rate and a G > A accelerator parameter from the data (a detailed description is provided in Methods). Points are colored by probability that the G > A accelerator parameter is >1. D. Phylogeny and highlighter plot of collapsed nucleotide variants. After collapsing by nucleotide sequence identity (Bubble size is scaled by the number of identical sequences), variants are numbered in descending order. The most frequent variant is used as the master sequence for the highlighter plot and as the root for the phylogeny. Any variant present at above 10 per cent of the population is colored in red on the tree. Colored lines in the highlighter plot represent different base substitutions or gaps (see key at the bottom of the panel).

The PORPIDpipeline was created to perform the large number of computational processes described in detail in Fig. 3, Supplementary Figure S3 and in the Methods section. In summary, PORPIDpipeline performs quality filtering, if not provided in the SMRT-UMI output, demultiplexing and UMI calling, filters out heteroduplexes, and performs contamination checks and consensus sequence generation, as well as initial sequence alignments. It also produces a report (Fig. 4) consisting of a summary of UMI family sizes and numbers, rejected sequences and the causes, hypermutations, a phylogenetic tree with identical sequences indicated by bubble size, and a highlighter plot. Core computational operations of the PORPIDpipeline were implemented in the Julia language for scientific computing and packaged into the Snakemake workflow management system.

### Example utilization of SMRT-sUMI in the study of virus biology

**Figure 5.**
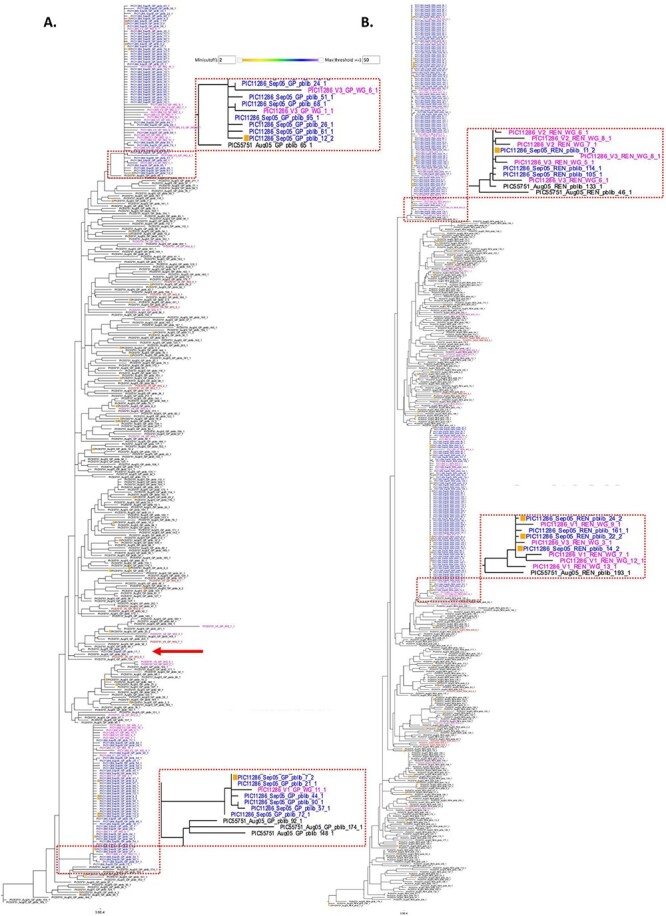
Deep sequencing of virus variants in an HIV transmission pair. SMRT-sUMI was used to assess viral variants transmitted in a known transmission pair previously investigated using Sanger NFLG sequencing. These correspond to transmission pair 4 from Herbeck et al. (2011) (transmitter PIC55751 and recipient PIC11286). Maximum likelihood trees (Deng et al. 2010) illustrating data from a 2.5 kb amplicon covering the gag gene (A) and a 3.1 kb amplicon covering the env gene (B) are shown as excerpts from Phylobook (Furlong et al., under review). Small colored boxes to the left of sequence names and numbers added as suffixes to the sequence name indicate that number of identical sequences collapsed into the sequence shown (see color key in inset). Sanger NFLG sequences are shown from the transmitter taken in the prior (Aug 2005, pink font) and subsequent (Oct 2005, red font) months following the recognition of infection in the recipient (three visits during Sep 2005, pink font). SMRT-sUMI sequences from the transmitter closest to the time of transmission (Aug05) are shown in black font and in blue font for the recipient. Expanded areas of the tree detail show the closest relatives between the two transmitted lineages and those found in the transmitter. The red arrow indicates the single sequence found in the 2.5 kb dataset corresponding to a third transmitted lineage. The scale bars at the bottom of the figures indicate a single nucleotide change.

To date, we have used SMRT-sUMI to sequence more than 500,000 individual HIV genome templates from more than 400 individuals, and at multiple time points. These numbers correspond to those passing the PORPIDpipeline with no irregularities noted. Approximately 90 per cent of the raw sequences from these studies passed the pipeline exclusion criteria. The enormous datasets generated have required development of additional bioinformatic tools to visualize and annotate this data, such as Phylobook (Furlong et al., submitted). Figure 5 shows an excerpt from a Phylobook entry with an example of the use of SMRT-sUMI to search in depth for viral variants that may have been transmitted in a known HIV transmission pair previously investigated using Sanger sequencing of approximately 10 near full length HIV genomes (NFLG, ∼9 kb) (Herbeck et al. 2011). In this example, the closest relatives to the recipient partner Sanger (pink font) and SMRT-sUMI (blue font) sequences found in the transmitting partner were SMRT-sUMI sequences (black font). Two distinct variant lineages, differing by up to 3.5 per cent, were transmitted to the recipient (see upper and lower blue clusters) that were evident in both 2.5 kb amplicons covering the gag gene and 3.1 kb amplicons covering the env gene. No recombinants between these two lineages were detected in the 3.1 kb amplicons (Fig. 5B, including 687 sequences). A single SMRT-sUMI sequence in the 525 sequence, 2.5 kb dataset (Fig. 5A) was detected outside of the two recipient clusters (red arrow). However, this sequence did not have signature sites indicative of recombination between the two major lineages but rather was closely related to another of the multiple lineages detected in the transmitting partner. These data illustrate both the low level or in this case the lack of artefactual recombinants produced in the SMRT-sUMI protocol and the detection of a third transmitted lineage that was not detected in prior studies.

## Discussion

In an effort to identify sUMI sample preparation methods allowing PCR amplification of diverse sequence populations while removing point mutational errors and minimizing the formation of artefactual recombinant molecules, we used a dUMI approach to track recombination events and a novel pipeline to produce matched sUMI and dUMI consensus sequences to compare sequence error rates. Relative to unpurified products, size selection using either bead or Blue Pippin purification after the first round of PCR resulted in greater recovery of the target amplicon, reduction of recombinant reads, and greater proportions of sequences passing 0.7 agreement filters, with the Blue Pippin method providing the best results. By reducing the number of cycles of the second round PCR by only 2, Blue Pippin purified samples with up to 277 templates per PCR resulted in fewer than 7 per cent identifiably recombinant reads. This highlights the importance of using as few cycles as possible to generate sufficient DNA for sequencing, as well as the ability to add more templates to each PCR if reducing cycle numbers. While reduced PCR cycles were not tested for all conditions, it can be reasonably assumed that reducing the number of cycles would have a similar effect on any preparation method as it reduces chances for recombination. While overall error rates were low when comparing sequences from sUMI and dUMI preparations, using fewer than 100 templates per PCR and either of the purification methods gave the lowest per base error rate. Ultimately, preparation methods cannot eliminate all forms of error or recombination events and so sUMI families must also pass the 0.7 filter to ensure high confidence in the consensus sequence. When applying the 0.7 filter, all sequences from samples with less than 100 templates per PCR were identical to the corresponding dUMI sequences, allowing use of only an sUMI. This avoids introduction of uncorrectable errors during the dUMI step, maximizes template recovery, and saves time and reagent expense. The 0.7 filter, along with many additional features for analyzing sequences prepared with the sUMI approach, such as summary tables and plots, as well as phylogenetic trees (Figs 3 and 4), are packaged together as the PORPIDpipeline.

In summary, balancing PCR template input against purification method and PCR cycle number is critical for limiting recombination events. The combination of limiting template input in PCRs to 100, using the fewest PCR cycles to result in sufficient material for sequencing (attaining sufficient material is easily achieved by conducting multiple parallel PCR reactions, each with limited numbers of templates), and performing a size selection after first round PCR, allows for highly accurate sequence determination of large genomic fragments using an sUMI, PacBio SMRT sequencing, and the PORPIDpipeline. This method allows for characterization of quasispecies at greater depths and gene lengths than previously possible by Sanger or short-read sequencing methods and provides a streamlined and generalized sequencing approach, which is highly adaptable to different pathogens and studies. These methods have been applied successfully to the different amplicons in this study plus many other studies of HIV (e.g. Corey et al. 2021; Juraska et al. 2024) and human herpesvirus 8 (Santiago et al. 2023) and in different laboratories to sequence multiple gene regions and with different levels of viral population diversity from >1000 samples across multiple large sequencing projects.

Alternative methods that are in some ways similar to SMRT-UMI have been reported. ClickSeq (Jaworski and Routh 2018) reduces error and recombination rates by replacing fragmentation and enzymatic ligation with chain termination and click-chemistry. However, it still relies on the amplification and sequencing of short fragments and the subsequent alignment of reads to create gene assemblies. SMRT-UMI sequencing methods can accurately sequence amplicons of any length that can be reliably amplified, allowing linkage of mutations across entire viral genes. The methods of Karst et al. (Karst et al. 2021) and HIV-Proviral UMI-mediated Long-read Sequencing (HIV-PULSE) (Lambrechts et al. 2023) and SMRT-UMI methods can amplify and sequence long fragments. A main advantage of sMRT-UMI over the method of Karst is the use of an sUMI, obviating the additional steps required to eliminate heteroduplexes and UMI-offspring errors. The main advantage of SMRT-UMI over HIV-PULSE is the lack of a pre-amplification step and therefore lack of a subsequent need for clustering of similar genomes. Indeed, the HIV-PULSE authors note that this clustering can impede identification of clonality. There are also fewer PCR and purification steps with the SMRT-UMI sUMI workflow. HIV-PULSE was developed to sequence proviral DNA, and, while SMRT-UMI methods were shown here to be used on HIV RNA, we also applied it to DNA templates by replacing the cDNA step with a single cycle of PCR utilizing an sUMI-containing primer (Santiago et al. 2023). HIV-PULSE also appears optimized for samples with low prevalence, such as proviral HIV, while SMRT-UMI methods and conditions are designed to accurately generate sequences from samples with any number of viral templates.

## Methods

### Specimen selection

Plasma specimens derived from four individuals (A-D) from the RV217 clinical cohort (Robb et al. 2016; Rolland et al. 2020) were used for these studies (Supplementary Table S1). The RV217 study cohort enrolled seronegative individuals in East Africa and Thailand; participants were tested twice weekly with an HIV-1 RNA test. Plasma samples collected in the first days after HIV-1 diagnosis from three participants from Thailand and one participant from Kenya were selected. The protocol was approved by the Walter Reed Army Institute of Research and local ethics review boards: the Walter Reed Project, Kericho, Kenya and the Armed Forces Research Institute of Medical Sciences, Bangkok, Thailand. Only adult participants were enrolled. Written informed consent was obtained from all participants. Institutional Review Board approval was obtained for use of the samples, and all were anonymized. Additional samples consisted of a transmission partner pair (Pair 4 from Herbeck et al. (2011) from the Seattle Primary Infection Clinic (Schacker et al. 1996)). Also sequenced were a virus stock and plasma from SIV-infected macaques, plasma from three individuals (M, N, and O) from the WIHS clinical cohort (Dapp et al. 2017), and two individuals infected with KSHV (Santiago et al. 2023).

### UMI primer composition

cDNA synthesis primers consisted of first round reverse primer-binding sequence (PB-R1-alt1—CCCGCGTGGCCTCCTGAATTAT), second round reverse primer binding sequence (PB-R2-alt1—CCGCTCCGTCCGACGACTCACTATA), 6-bp Sample ID sequence, 8-bp random UMI sequence, and a template binding sequence (Fig. 1A). Second strand synthesis primers consisted of the first round forward primer binding sequence (illu_F1—AATGATACGGCGACCACCGA), second round forward primer binding sequence (illu_F2—GATCTACACTCTTTCCCTACACG), 8-bp random UMI sequence, and a template binding sequence. All primers were synthesized by Integrated DNA Technologies and those containing a UMI sequence were synthesized with the ‘Hand-Mix’ option selected for the 8-bp random UMI sequence. Subsequently, the use of primers without the Hand-Mix option were validated. To allow pooling of multiple samples during sequencing, a different 6-bp Sample ID was used for each cDNA primer. All primer sequences and usage are listed in Supplementary Table S2.

### Library amplification and sequencing

Preparation of dUMI samples from KSHV DNA in tumor biopsies has been described previously (Santiago et al. 2023). Preparation of all other sUMI and dUMI samples is described later. RNA was extracted from plasma using the QIAamp Viral RNA Mini Kit (QIAGEN) and then used as template for cDNA synthesis, PCR and sequencing. About 50 μl cDNA synthesis reactions were performed using Superscript III Reverse Transcriptase (Thermo Fisher Scientific) supplemented with 1 U ThermaStop-RT (Sigma-Aldrich) per 50 U SSIII to increases RT-PCR specificity. One microliter of SSIII was added after 1.5 h and incubated for another 1.5 h to increase recovery of rare templates; however, similar results were obtained using SSIV with a 1-h incubation. RNA was then removed by incubation with 2 U RNase H [2 U/μl, Invitrogen] for 20 min at 37 °C. Each cDNA synthesis reaction utilized UMI-containing primers with a sample ID. To remove unincorporated primers, cDNA was purified with RNAClean XP magnetic beads (Beckman Coulter) at a 1:1 ratio with three 80 per cent ethanol washes and eluted into 25 µl of nuclease-free water. In dUMI preparations, 25 μl of purified cDNA was subjected to second strand cDNA synthesis in a 40 μl reaction using 0.8 μl PrimeSTAR GXL (Takara Bio), 8 μl 5× Buffer, 3.2 μl dNTP Mix (2.5 mM), and 10 pmol of a second strand primer mix containing UMI sequences. Reaction conditions were: 2 min at 98 ^o^C, 30 s at 60 ^o^C, 10 min at 68 ^o^C and a 4 ^o^C hold. Second strand product was purified with AMpure XP magnetic beads in the same manner as the cDNA and eluted into 40 μl of nuclease-free water.

The concentration of amplifiable templates in purified cDNA or second strand products was estimated by performing 25 μl nested PCRs with 0.5 μl PrimeSTAR GXL, 5 μl 5× Buffer, 2 μl dNTP Mix (2.5 mM), and 10 pmol of forward and reverse primers on limiting dilutions of the products then inputting the number of positive PCR per dilution to the web program Quality [https://indra.mullins.microbiol.washington.edu/quality/] (Rodrigo et al. 1997). First round PCR cycling conditions (35 cycles) were: 2 min at 98 ^o^C; 35 cycles of 10 s at 98^o^ C, 15 s at 60 ^o^C, and 1 min/kb extension at 68 ^o^C; followed by a 7 min extension at 68 ^o^C and a 4 ^o^C hold. Second round PCR cycling was identical except that an annealing temperature of 62 ^o^C was used instead of 60 ^o^C. The pNL4-3 plasmid (Adachi et al. 1986) was used as template in a positive control reaction with a template-specific reverse primer spiked in, as this plasmid does not contain the primer binding sites incorporated by the UMI containing primers.

Using the estimated copy number results from the Quality output, 25 μl first round PCRs were set up with an estimated 25, 50, or 100 UMI-labeled templates per reaction for Participants A, B, and C and 25 or 200 for Participants E and F. All other samples prepared with only one input, usually limited to twenty-five or fewer templates per reaction by the concentration of target templates in the sample. The number of identical first round reactions varied by sample from 1 to 18 with the goal of sequencing at least a total of 100 templates per preparation. First round amplification conditions were the same as described for the template estimation reactions except that only 20 cycles of amplification were performed.

For samples comparing purification methods, first round PCR products were pooled and mixed. About 20 μl of each pool was diluted in 40 μl of 10 mM Tris-Cl, pH 8.0 to normalize across samples and ensure sufficient volume to test each method. Thirty microliters of this volume was purified by size selection on a Blue Pippin instrument (Sage Science) using a 0.75 per cent agarose cassette with Low Voltage 1–6 kb definition and marker S1 on the Tight mode setting, using the size of the first round amplicon as the target. In parallel, 20 μl of the diluted volume was purified with AMpure XP magnetic beads at 0.5× ratio, washed 3 times with 80 per cent ethanol (EtOH), and eluted into 26.7 μl of 10 mM Tris-Cl, pH 8.0. Lastly, 5 μl unpurified product diluted with 1.67 μl 10 mM Tris-Cl, pH 8.0 to match the dilution effects of the purification methods. About 30 μl of undiluted first round was purified using Blue Pippin for samples without a purification comparison.

One microliter of pooled Blue Pippin, Ampure Bead or unpurified fractions was used as template in a single 25 μl second round PCR. Conditions were the same as described for the template estimation reactions except that cycle number (20–22) varied by sample due to differences in amplification efficiencies and number of starting templates per PCR (Supplementary Table S1). From individuals A–C, Blue Pippin purified fractions from the 100 estimated copy samples were also used as template in two second round PCR, one with 22 cycles, and one with only 20 cycles of amplification to directly test the effects of reducing cycle numbers. Twenty microliters of each second round PCR was purified with AMpure XP magnetic beads at 0.6× ratio followed by three 80 per cent EtOH washes and eluted into 20 μl of 10 mM Tris-Cl, pH 8.0.

In samples comparing purification methods in parallel, each sample from a given subject contained the same Sample ID, so an additional round of PCR was performed to incorporate a unique pair of index primers for each sample. About 5 μl of purified second round PCR product was used as template in a 50 μl PCR with PrimeSTAR GXL containing a unique combination of one forward and one reverse custom or index primer or one forward and one reverse Illumina Nextera Index primer (Supplementary Table S2). PCR conditions were the same as described earlier but with an altered cycling protocol: 2 min at 98 ^o^C; 5 cycles of 10 s at 98 ^o^C, 30 s at 55 ^o^C, and 1 min/kb extension at 68 ^o^C; followed by a 7 min extension at 68 ^o^C and a 4 ^o^C hold.

The optimized sUMI protocol utilizes four second round PCRs per sample, with 20 cycles of amplification, and does not require an extra PCR to incorporate index primers. Instead, a single cycle of synthesis and extension with fresh ingredients was performed to eliminate heteroduplexes. For this, 5 μl of the following mixture was spiked into each second round PCR, 0.5 μl PrimeSTAR GXL, 1 μl 5× Buffer, 2 μl dNTP Mix (2.5 mM), and 10 pmol of forward and reverse primers, and incubated for 2 min at 98 ^o^C, 15 s at 62 ^o^C, and 10 min at 68 ^o^C.

Second round PCR products were purified with AMpure XP magnetic beads at a 0.6× ratio, washed 3 times with 80 per cent EtOH and eluted into 40 μl of 10 mM Tris-Cl, pH 8.0. Concentrations of purified DNA were determined using a Qubit dsDNA HS Assay Kit (ThermoFisher). Samples were combined in equimolar amounts with no duplicate Sample IDs or index combinations in the same pool of samples, then purified with AMpure XP magnetic beads at 0.7× ratio, washed 3 times with 3 × 80 per cent EtOH and eluted into 40 μl of 10 mM Tris-Cl, pH 8.0. The concentration of pools combined for sequencing on the same SMRT cell was also determined using Qubit. Library preparation was performed on each pool using the SMRTbell Express Template Prep Kit 2.0 (PacBio) with each pool receiving a different barcoded adapter from the Barcoded Overhang Adapter Kit–8A (PacBio). Completed libraries were purified with SMRTbell enzyme cleanup kit to remove incomplete or damaged SMRTbell molecules and then sequenced on a SMRT Cell 8 m 15-h movie using the Sequel II instrument (PacBio). Number of samples per pool and number of pools per SMRT cell depended on number of estimated templates per sample, where total estimated templates across all samples and pools were ∼20,000–40,000 per SMRT cell.

Sanger SGA amplification and sequencing were described previously for Participant B (Rolland et al. 2020), Participants M–O (Corey et al. 2021), Participant T (Corey et al. 2021), and PIC participants PIC11286 and PIC55751 (Herbeck et al. 2011).

### Bioinformatic processing

Three snakemake (Molder et al. 2021) pipelines were created; one that performed the basic function of demultiplexing by index primer (chunked_demux), one that performed additional demultiplexing by sample ID, UMI identification, and consensus sequence generation for sUMI and dUMI read collections (sUMI_dUMI_comparison) to test the different preparation methods, and one for use with the optimized sUMI approach with additional functionality after the consensus step (PORPIDpipeline). Because the sample preparations were labeled with index primers, the additional demultiplexing step (chunked_demux) was necessary to bin the reads into separate fastq collections by index combination and remove index primer sequences. These collections were then used as input for the sUMI_dUMI_comparison pipeline where standard demultiplexing using the Sample ID took place.

All three pipelines are provided as open source via Github (Murrell 2022b; Murrell,Pankow, and Westfall, 2023; Pankow and Westfall, 2023). Snakemake decomposes the workflow into rules that define how to obtain the stated output files from the given input files. The Snakefile lists the input and output files for each rule as well as user-defined parameters and the shell script that executes this process. Additionally, a user-supplied config file lists each sample to be processed, along with each sample’s corresponding primer sequences and/or reference panels. A description of each rule in PORPIDpipeline is shown in Fig. 3 and described in more detail later. Only the early versions of the demux, porpid, and consensus rules were present in the sUMI_dUMI_comparison pipeline.

#### Demux

##### Quality Filtering and Demultiplexing.

After sequencing, PacBio raw sequence data were processed to produce CCS, which are provided to the pipeline as fastq files (optionally gzipped). Each CCS is first examined and rejected if the mean error rate, inferred from the fastq quality scores (Laird Smith et al. 2016), is greater than the user-supplied parameter (default 0.01). Any CCS with length above or below user-supplied parameters are also rejected. Each CCS is then assigned to a sample according to the PCR primers and Sample IDs listed in the config file, tolerating end degradation, and some imperfect matching on the primers, but requiring complete matching on the Sample IDs.

#### Porpid

##### Probabilistic Offspring Resolver for Primer IDs.

For each sample, PORPID uses an alignment with the primer sequence to identify the UMI barcode in each CCS sequence, and groups sequences, by UMI, into ‘UMI families’. Since sequencing errors in the UMI itself can create erroneous ‘offspring’ UMI barcodes, PORPID uses a probabilistic model (Supplementary Fig. S3) that considers the probability of generating each observed UMI from each other UMI by error and then infers the underlying UMI frequencies from the noisily observed UMIs. UMI families that are likely to be erroneous, which are typically low-frequency neighbors of higher-frequency real UMI families, are excluded from downstream processing. Each UMI family is then examined and those that have UMI of length not equal to 8 or CCS number fewer than 5 are excluded.

Next, UMIs produced as a result of heteroduplexes are removed. During PacBio SMRT sequencing, each strand of the circularized DNA template is successively sequenced. As the polymerase synthesizes each strand in a UMI library derived from a heteroduplex, two different UMI sequences will be read. This phenomenon produces a drop in PHRED scores (Ewing et al. 1998; Ewing and Green 1998) for the UMI bases after circular consensus generation, since the two strands disagree, and the algorithm identifies this disagreement using a combination of heuristic rules and statistical comparison to other regions. For the sUMI_dUMI_comparison pipeline, all UMI-2 sequences on the second strand were recorded but not subjected to the filtering used for the UMI-1 sequence as described earlier (Table 1).

#### Consensus

##### Consensus Sequence Generation.

Consensus sequences for UMI families that pass all quality control criteria are generated as described (Kumar et al. 2019; Murrell 2022a). Briefly, an exemplary read is selected based on the kmer distributions of each read, compared to the average for that UMI family, and then refined by alignment of all reads, modifying the consensus where the majority of reads mismatch. Read agreement to the final ‘polished’ consensus is calculated and used downstream for the minimum agreement filter. The family size and minimum agreement values are reported for each consensus sequence and written as FASTA.

#### Contam

##### Contamination Filter.

A rapid contamination screen is used, based on the observation that, for closely-related sequences, a kmer-derived distance well approximates an edit distance (Kumar et al. 2019). Consensus sequences for the same sample are subjected to kmer-vector clustering using a 1.5 per cent radius. An overall centroid for each sample is merged with centroids for clusters making up more than 20 per cent of the sample population and combined with kmer representations of common lab contaminants stored by the user in a supplied FASTA file. Default settings are such that sequences within a 1.5 per cent distance from another cluster centroid and >1.5 per cent away from their own cluster centroid are discarded. Sequences that are outside a 1.5 per cent corrected kmer vector distance from another cluster centroid and <1.5 per cent away from their own cluster centroid are retained. Summary information is supplied to the user as a csv file.

#### Postproc

##### Post-processing Steps.

For each sample, sequences that survived the contamination check are filtered using the minimum agreement values. This is a user supplied parameter but suggested to remain at default of 0.7. Insertions at the ends of the sequences are trimmed off via profile alignment to a user supplied reference panel. Major misalignments such as off-target sequences (≥50 per cent different from profile of a reference panel) are excluded. Outputs from postproc include the filtered sequence collection, mafft (Katoh and Standley 2013) alignment of these sequences, maximum likelihood tree, and a highlighter plot of collapsed sequences. Also produced are additional summary plots to be used by the Report rule.

#### Report

HTML reports are generated for each sample (Fig. 4). These contain a strip-plot indicating family sizes and a table summarizing rejected CCS, as well as two plots which help to provide preliminary information about sequences and lineages: a maximum likelihood tree and highlighter plot of collapsed sequences, and a 2D projection of the sequences by multidimensional scaling, colored by the probability of an inflated G > A mutation rate using a modified nucleotide substitution model. A Bayesian approach was used to highlight sequences that are most likely generated through APOBEC-mediated hypermutation. Starting from a set of sequences belonging to an individual sample, identification of individual sequences with an inflated G to A mutation rate was performed as follows. Given a query sequence *S* and the consensus *C* for the sampled population, evolution of each sequence from *C* to *S* is considered as a continuous-time Markov chain. While this choice of ancestor is an oversimplification, *C* is easily calculable and gives a reasonable estimate of the rates of base substitutions for our purposes. For the rate matrix, we used a modified K80 2-parameter model (Kimura 1980) where in addition to the transition-to-transversion rate ratio we included an accelerator parameter for G to A transitions.

Specifically, the rate matrix $Q$ is defined as:


$$Q = \left( {\begin{array}{*{20}{c}}
{\mathrm{*}}&1&\kappa &1\\
1&{\mathrm{*}}&1&\kappa \\
{\kappa \gamma }&1&{\mathrm{*}}&1\\
1&\kappa &1&{\mathrm{*}}
\end{array}} \right)$$

with columns corresponding to *A, G, C*, and *T*, respectively. Given the observed sequence substitutions between *C* and *S* and $\kappa $ set to 4.5, the posterior probability distribution of the rate *t* and accelerator $\gamma $ are estimated computationally using a Bayesian approach. The calculation is done via a discretization of the two parameters, providing a grid of points at which the posterior is evaluated. For the prior distribution over *γ*, we wished to allow a small proportion of sequences to have a wide range of possible G to A mutation rates, so we used a mixture of two Gaussians. We define the prior $P\left( {{\mathrm{log}}\gamma } \right)$ as


$$P\left( {{\mathrm{log}}\gamma } \right) \sim {\omega _1} \cdot N\left( {\mu ,{\sigma _1}} \right) + {\omega _2} \cdot N\left( {\mu ,{\sigma _2}} \right)$$

where, ${\omega _1} = 0.99$, ${\omega _2} = 0.01$, ${\sigma _1} = 0.1$, ${\sigma _2} = 1$, and $\mu = 0$ all in the log domain. For the visualization, we colored sequences by the posterior probability that $\gamma > 1$. All code are available in the *PORPIDpipeline* repository at src/apobec_model.jl.

#### Index

An HTML index page is created containing summary tables and links to each sample report.

#### Tar

The porpid and postproc directories are archived and gzipped ready for downstream processing.

Compressed input/output files, config files, code written and executed in python and R studio, and sequence alignments were uploaded to the Dryad and Zenodo databases upon submission (https://doi.org/doi:10.5061/dryad.w3r2280w0) with a README file and Analysis Flowchart (Supplementary Fig. S4) to indicate which files originate from each step and where they were used in the overall analysis. The custom code written for this analysis was used to collect and combine output files containing UMI information, calculate LD (van der Loo 2014), perform recombination analyses, and write out data tables for plotting figures with Prism software (GraphPad). Figure 2D used a smoothing spline fit (four knots) to generate fits. A python script was used to compare matched consensus sequence sets, all diversity measures were calculated using DIVEIN (Deng et al. 2010), as a plugin for Phylobook (Furlong et al. under review), and all alignments were created in Geneious software (BioMatters).

## Supplementary Material

veae019_Supp
