# Correction to: Optimized SMRT-UMI protocol produces highly accurate sequence datasets from diverse populations—Application to HIV-1 quasispecies

**DOI:** 10.1093/ve/veae038

**Published:** 2024-05-14

This is a correction to: Dylan H Westfall, Wenjie Deng, Alec Pankow, Hugh Murrell, Lennie Chen, Hong Zhao, Carolyn Williamson, Morgane Rolland, Ben Murrell, James I Mullins, Optimized SMRT-UMI protocol produces highly accurate sequence datasets from diverse populations—Application to HIV-1 quasispecies, Virus Evolution, Volume 10, Issue 1, 2024, veae019, https://doi.org/10.1093/ve/veae019.

When this article originally published, author Hugh Murrell’s name was omitted from the author list, due to an error which had occurred during the production process.The Publisher apologizes for this.The article has been corrected.

