# Supplementary figures and images for: Optimized SMRT-UMI protocol produces highly accurate sequence datasets from diverse populations—Application to HIV-1 quasispecies
Source: Virus Evol. 2024 Mar 2;10(1):veae019. doi: 10.1093/ve/veae019 (PMC11099545; doi:10.1093/ve/veae019)

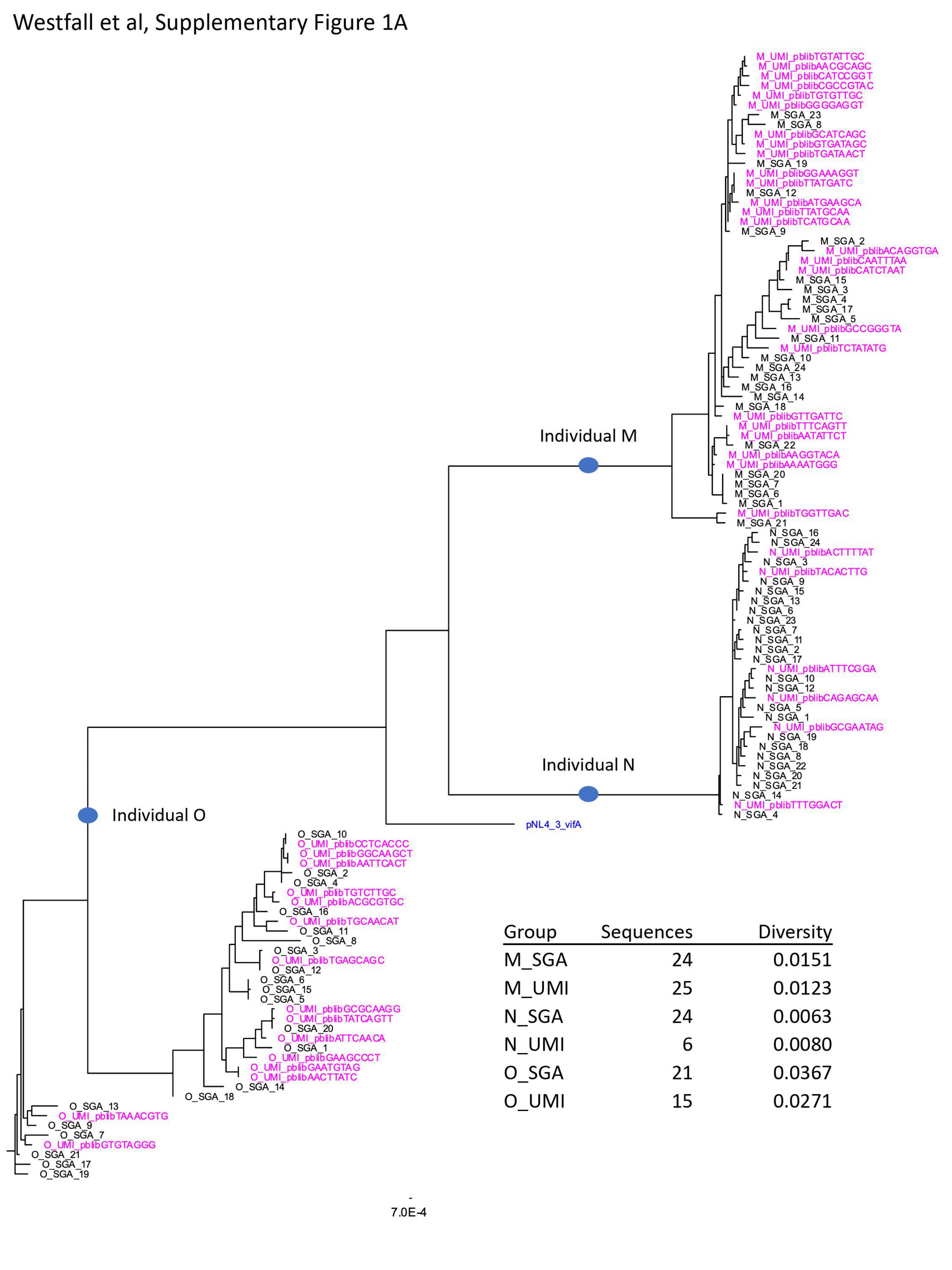

Supplement: veae019_Supp [file veae019_supp.zip › suppl_data/DWestfall_PacBioMethods_Feb2024_SupFig1a.jpg]

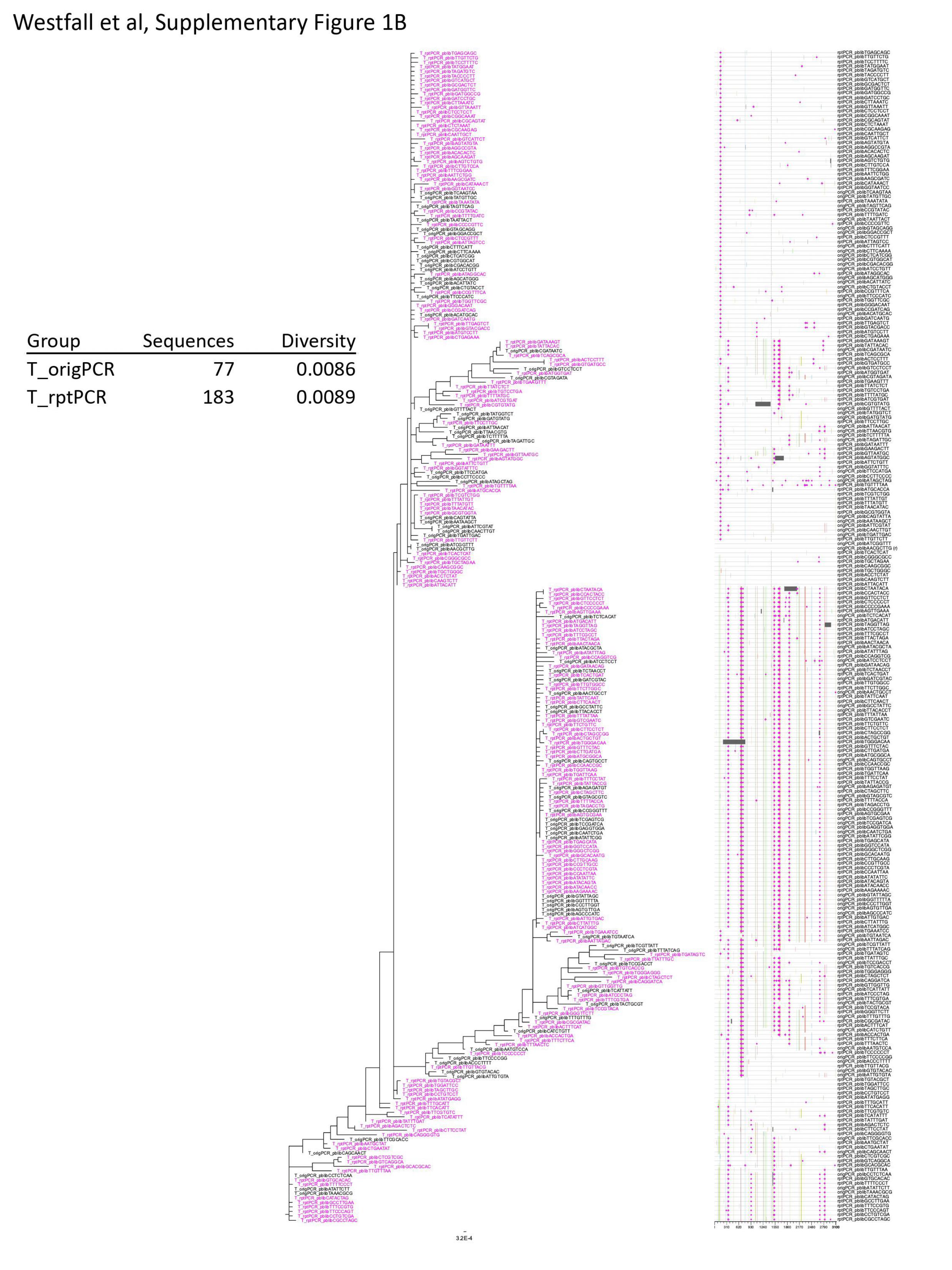

Supplement: veae019_Supp [file veae019_supp.zip › suppl_data/DWestfall_PacBioMethods_Feb2024_SupFig1b.jpg]

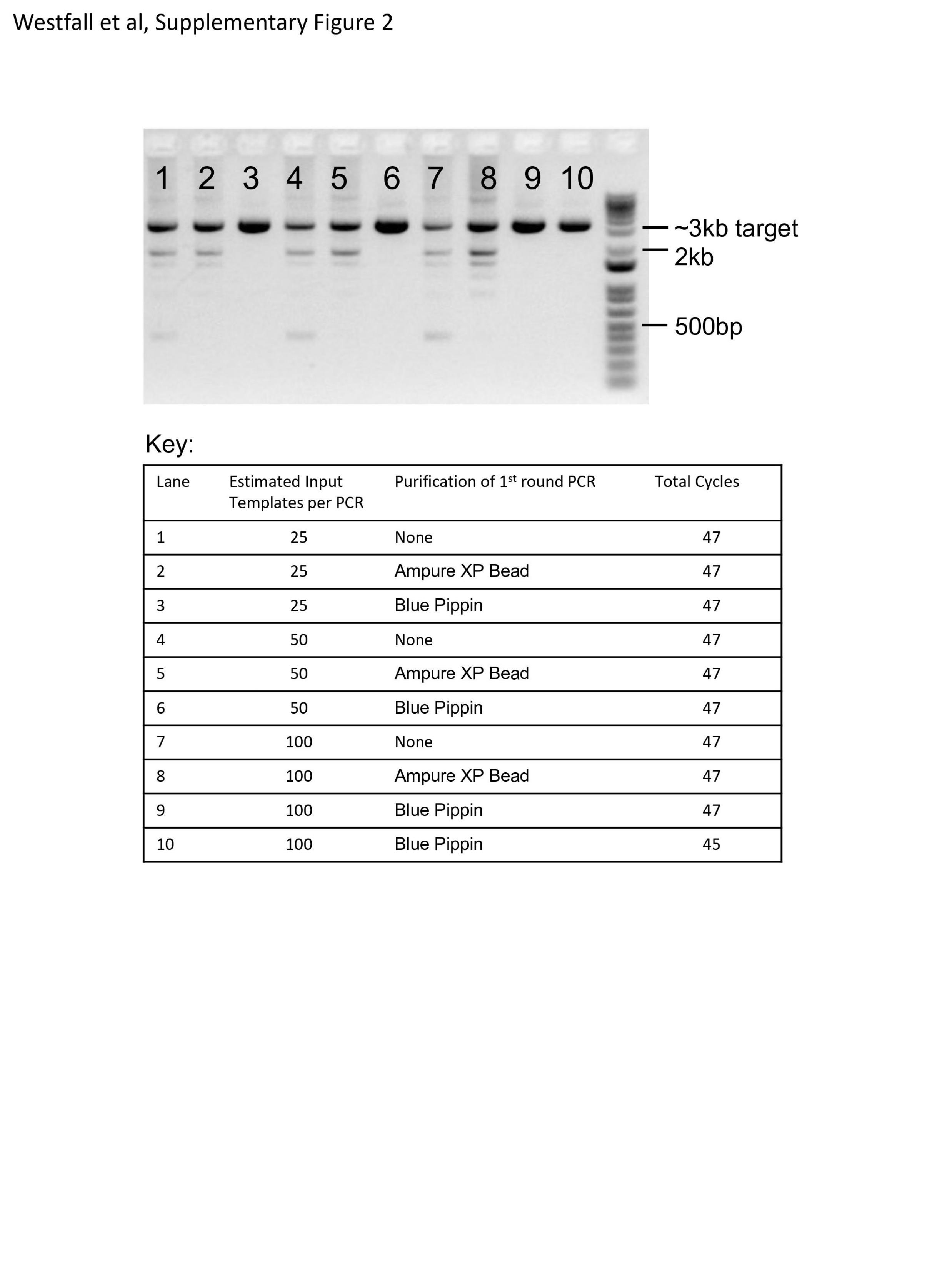

Supplement: veae019_Supp [file veae019_supp.zip › suppl_data/DWestfall_PacBioMethods_Feb2024_SupFig2.jpg]

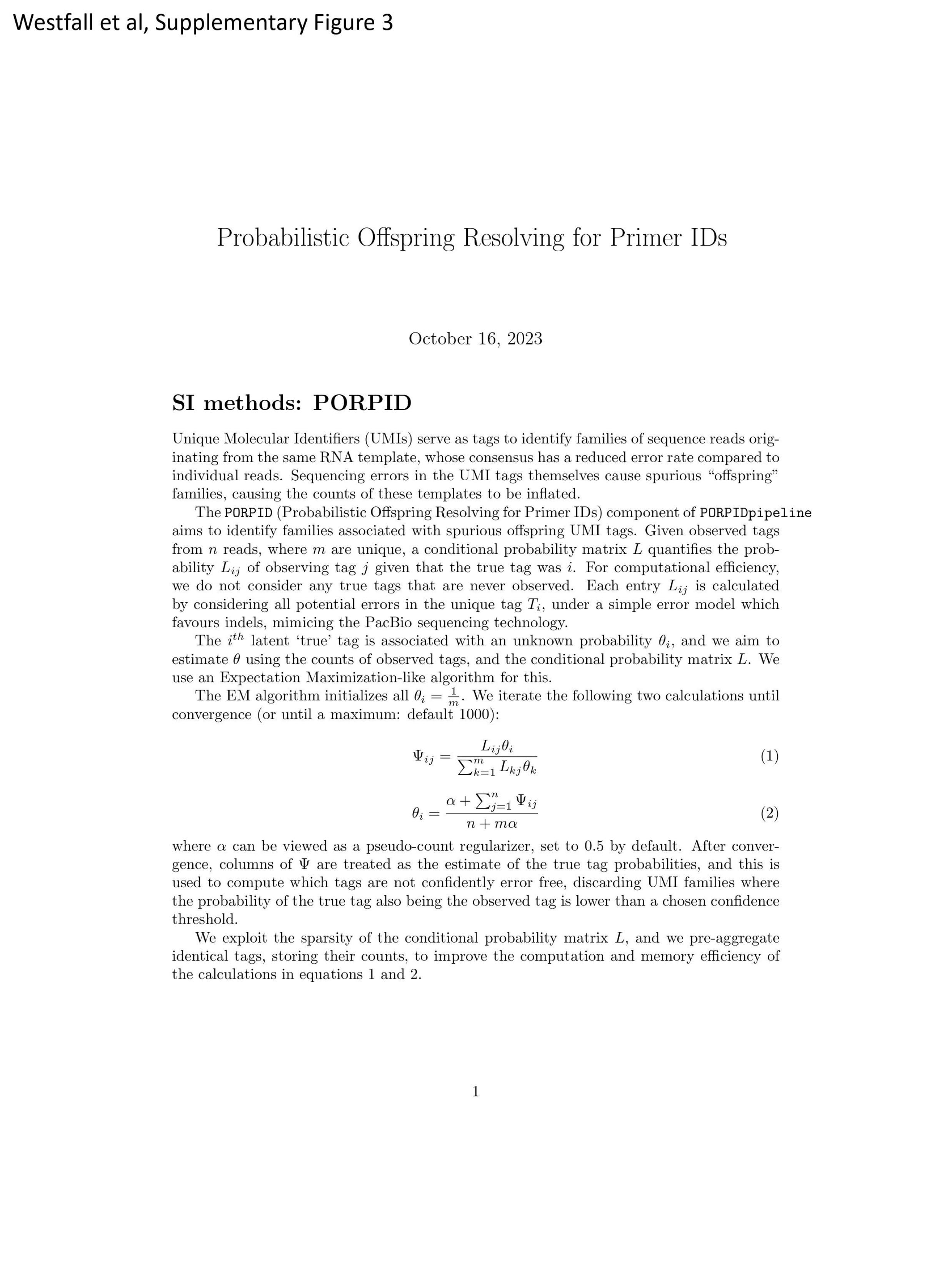

Supplement: veae019_Supp [file veae019_supp.zip › suppl_data/DWestfall_PacBioMethods_Feb2024_SupFig3.jpg]

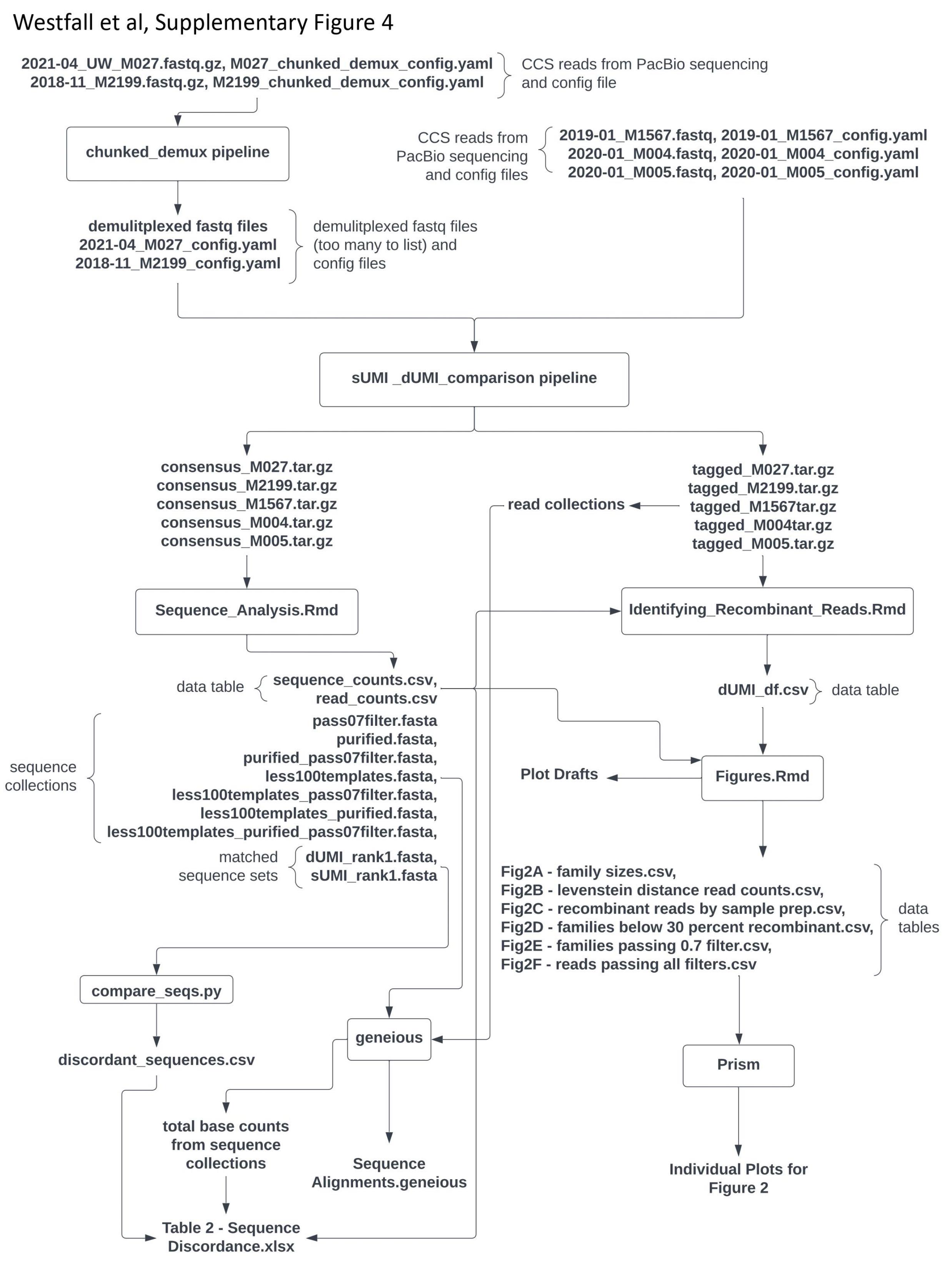

Supplement: veae019_Supp [file veae019_supp.zip › suppl_data/DWestfall_PacBioMethods_Feb2024_SupFig4.jpg]
